# Branched Amphiphilic Polylactides as a Polymer Matrix Component for Biodegradable Implants

**DOI:** 10.3390/polym15051315

**Published:** 2023-03-06

**Authors:** Vladislav Istratov, Vitaliy Gomzyak, Valerii Vasnev, Oleg V. Baranov, Yaroslav Mezhuev, Inessa Gritskova

**Affiliations:** 1 A.N. Nesmeyanov Institute of Organoelement Compounds of Russian Academy of Sciences, Vavilov Street, 28, 119991 Moscow, Russia; 2 Bauman Moscow State Technical University, Baumanskaya 2-ya Str., 5/1, 105005 Moscow, Russia; 3 Department of Chemistry and Technology of Macromolecular Compounds, MIREA—Russian Technological University (RTU MIREA), Vernadskogo Avenue 78, 119454 Moscow, Russia; 4 Department of Biomaterials, Mendeleev University of Chemical Technology of Russia, Miusskaya Sq., 9, 125047 Moscow, Russia

**Keywords:** amphiphilic block copolymers, polylactide, thermal properties, mechanical properties, hydrophilicity, hydroxyapatite

## Abstract

The combination of biocompatibility, biodegradability, and high mechanical strength has provided a steady growth in interest in the synthesis and application of lactic acid-based polyesters for the creation of implants. On the other hand, the hydrophobicity of polylactide limits the possibilities of its use in biomedical fields. The ring-opening polymerization of L-lactide, catalyzed by tin (II) 2-ethylhexanoate in the presence of 2,2-bis(hydroxymethyl)propionic acid, and an ester of polyethylene glycol monomethyl ester and 2,2-bis(hydroxymethyl)propionic acid accompanied by the introduction of a pool of hydrophilic groups, that reduce the contact angle, were considered. The structures of the synthesized amphiphilic branched pegylated copolylactides were characterized by ^1^H NMR spectroscopy and gel permeation chromatography. The resulting amphiphilic copolylactides, with a narrow MWD (1.14–1.22) and molecular weight of 5000–13,000, were used to prepare interpolymer mixtures with PLLA. Already, with the introduction of 10 wt% branched pegylated copolylactides, PLLA-based films had reduced brittleness, hydrophilicity, with a water contact angle of 71.9–88.5°, and increased water absorption. An additional decrease in the water contact angle, of 66.1°, was achieved by filling the mixed polylactide films with 20 wt% hydroxyapatite, which also led to a moderate decrease in strength and ultimate tensile elongation. At the same time, the PLLA modification did not have a significant effect on the melting point and the glass transition temperature; however, the filling with hydroxyapatite increased the thermal stability.

## 1. Introduction

One of the most urgent problems of bioengineering is the development of new biodegradable polymeric devices. Despite intensive research in recent decades, the number of polymers available for this application is extremely limited. One of their most prominent representatives is poly(L-lactide) (PLLA). It is an aliphatic semi-crystalline polyester, that can be produced from renewable sources such as corn or sugar cane [1]. Due to its biocompatibility, biodegradability up to lactic acid, and sufficiently high mechanical characteristics, as well as a biodegradation rate comparable in time to the healing of damaged human tissues, products made from this polymer are of great interest for use in regenerative medicine [2,3,4,5]. Unfortunately, due to the fragility and hydrophobicity of PLLA [6], its use in medicine is limited [7]. One possible way to vary the properties of PLLA devices, including the rate of their biodegradation, is to manufacture these devices from PLLA plasticized with other polymers [8,9,10,11,12]. A promising component of PLLA matrices for biodegradable devices are amphiphilic copolyesters, in most cases represented by block copolymers of polylactide or polycaprolactone with polyethylene oxide [13,14,15,16]. They have been used to create capable biomedical devices such as stents, films, thermosensitive polymer hydrogels, electrospun fibers, etc. [17,18,19,20,21,22].

Despite the wide variety of polymers used to modify PLLA, the vast majority of them have a linear structure. At the same time, in recent decades [23,24,25,26,27], interest has been growing in the synthesis and study of the properties of biocompatible, biodegradable, branched and hyperbranched polymers, the properties of which differ significantly from linear, star-shaped, and crosslinked analogs. As a rule, their main feature is a smaller size of molecules compared to linear analogs, a higher density of the structure of macromolecules, in combination with a spatially unloaded core, as well as a large number of free functional groups located on the surface [28,29,30]. All these types of macromolecular substances differ significantly in properties from their linear counterparts, and their main feature lies in the possibility of consistent regulation of the structure and, accordingly, properties. From this point of view, branched and hyperbranched polymers are of great interest; by modifying their free reactive functional groups, one can change the properties of polymers over a very wide range [31,32,33,34], and obtain biodegradable amphiphilic copolymers for targeted drug delivery [35,36,37,38]. An additional modification of functionalized polymers makes it possible to obtain copolymers with controlled colloidal chemical properties [39,40]. The use of amphiphilic branched polymers as a component of the polymer matrix in hydroxyapatite-containing systems also leads to a significant improvement in the properties of composite materials. In these systems, branched amphiphilic polylactides act not only as PLLA plasticizers, which change the mechanical properties of the polymer matrix, but also as compatibilizers, which increase adhesion between the polymer matrix and the mineral filler. The latest advances in the field of regulation of mechanical properties, and the use of branched polylactides in biomedical fields, were summarized in review [41].

Ring-opening polymerization of lactones (ROP), catalyzed by tin carboxylates in the presence of dihydroxycarboxylic acids as co-initiators and branching agents of the AB2 type, were discussed in the literature in connection with the synthesis of branched copolyesters [42,43,44,45]. Kinetic data indicate that ROP proceeds via the sequential insertion of lactones into the –Sn–OR bond, after the formation of tin alcoholates from the starting tin carboxylates [42]. Bis(hydroxymethyl)butyric acid is traditionally used as an AB2 agent [44,45].

**Figure 1:**
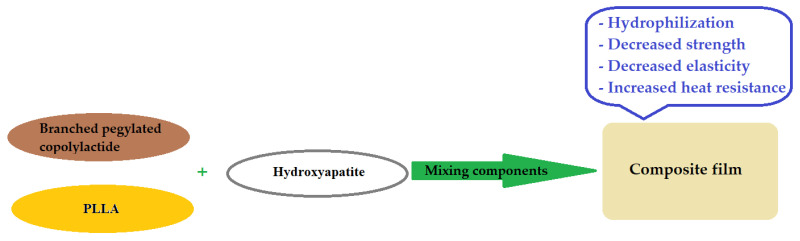
The preparation and development of properties of polylactide composite materials filled with hydroxyapatite.

In the present paper, in order to introduce long-chain hydrophilic fragments and reduce the wetting angle of the surface of the resulting branched copolylactides, an ester obtained by esterification of 2,2-bis(hydroxymethyl)-propyonic acid and poly(ethylene glycol) monomethyl ethers, was used as a macroinitiator. The synthesized pegylated branched copolylactides were compatible, when mixed with linear PLLA, providing its hydrophilization, which also contributed to the good compatibility of the prepared interpolymer mixtures with a hydrophilic filler, i.e., hydroxyapatite. The filling of polylactide interpolymer blends with hydroxyapatite contributed to an additional decrease in the water contact angle; however, this effect was achieved at the cost of a decrease in strength and ultimate tensile elongation (Figure 1). At the same time, the mechanical characteristics of the obtained materials were acceptable for use in biomedical fields, in particular, for bone tissue regenerative surgery.

## 2. Materials and Methods

### 2.1. Materials

Polylactic acid (PLLA, Mw ~60 kDa, Aldrich Chemical Co., St. Louis, MO, USA), stannous (II) 2-ethylhexanoate (Sn(Oct)_2_, 95%, Aldrich Chemical Co., St. Louis, MO, USA), 1,1′-carbonyldiimidazole (CDI, 98%, Aldrich Chemical Co., St. Louis, MO, USA), and hydroxyapatite (HA, particle size ~1 micron, “R&P” “POLYSTOM”, Moscow, Russia) were used as received. L-lactide (98%, ABCR, Karlsruhe, Germany) was recrystallized from ethyl acetate before use; 2,2-bis(hydroxymethyl)propyonic acid (BHP, 98%, Aldrich Chemical Co., St. Louis, MO, USA), poly(ethylene glycol) monomethyl ethers, with Mn ~1900 and 5000 Da (MPEG1900 and MPEG 5000, respectively, Merck, Darmstadt, Germany) were dried in a vacuum (10 Pa, 30 °C) for 24 h prior to use. Methylene chloride, THF, and DMSO (“Himmed”, Moscow, Russia) were purified by standard methods [46].

### 2.2. Polymerization

**Scheme 1:**
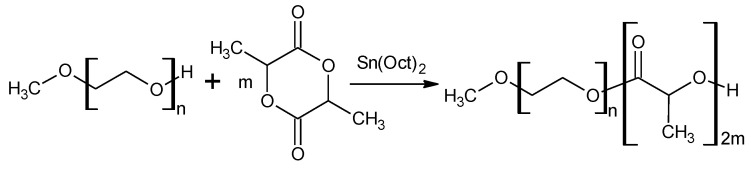
Synthesis of linear copolylactide.

Linear copolylactide was prepared by copolymerizing L-lactide and MPEG 1900; synthesis was carried out at 140 °C for 24 h in bulk, using Sn(Oct)_2_ as a catalyst (molar ratio L-lactide:catalyst = 2000:1) according to Scheme 1 [47].

**Scheme 2:**
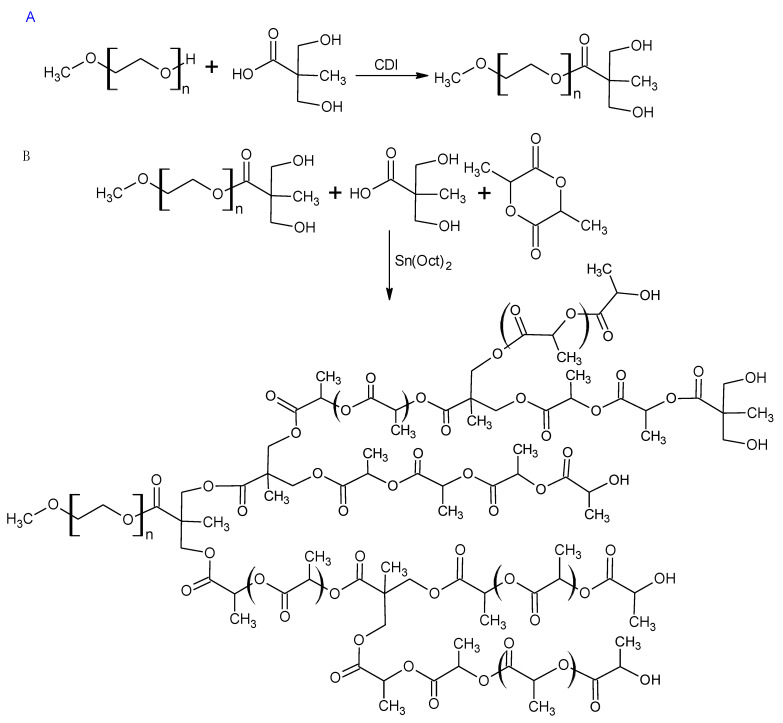
Synthesis of pegylated branched copolylactides: esterification of BHP upon interaction with MPEG (**A**); polymerization of L-lactide in the presence of BHP and BHP ester with MPEG (**B**).

Branched copolylactides were prepared by ROP of L-lactide in the presence of BHP, as well as the esterification product of BHP and MPEG [48,49], as per Scheme 2.

Thus, to obtain linear copolylactide 1, the Sn(Oct)_2_ catalyst (0.008 g, 0.01 mmol) was placed in a two-necked glass flask in the form of a 10% solution in THF, after which the solvent was distilled off under reduced pressure. Then, 2.883 g, 20.0 mmol of L-lactide and 0.150 g, 0.40 mmol of MPEG 1900 were added to the flask. The flask was equipped with a mechanical overhead stirrer and heated to 140 °C using a heating mantle, for 24 h. The resulting crude product was dialyzed in CHCl_3_ (Roth “ZelluTrans” membrane, MWCO = 1000 Da, Karlsruhe, Germany) for 48 h. Then, the solvent was evaporated and the polymer was dried under vacuum for 48 h [47].

For the synthesis of branched copolylactides in the first stage (Scheme 2A), MPEG (10 mmol) and CDI (16.21 g, 10 mmol) were reacted in 50 mL of THF, for 30 min at room temperature, after which this solution was slowly added to BHP (14.75 g, 11 mmol) in 50 mL THF, at 30 °C, and reacted for 2 h. The resulting crude product was purified by column chromatography on silica gel (eluent—THF) with a yield of 68%. At the next stage, an ester of BHP and MPEG was introduced into copolymerization with L-lactide and BHP (Scheme 2B, Table 1). The reaction was carried out at 140 °C, as described in article [47]. The resulting crude product was dialyzed in CHCl_3_ (Roth “ZelluTrans” membrane, MWCO = 1000 Da, Karlsruhe, Germany) for 48 h. Then the solvent was evaporated and the polymer was dried under vacuum for 48 h [47].

### 2.3. Characterization

The ^1^H NMR spectra were recorded on a Brucker spectrometer, with operating frequency ^1^H—400.22 MHz at 25 °C, using CDCl_3_ as solvent and TMS as an internal standard.

FTIR spectra were recorded on a Nicolet 380 spectrometer (Thermo Fisher Scientific Inc., Waltham, MA, USA).

Size exclusion chromatography (SEC) of the copolymers was carried out on a “Waters 150” chromatograph (eluent—THF (1 mL/min), column—PL-GEL 5u MIXC (300 mm × 7.5 mm) (“Waters”, Milford, MA, USA).

The differential scanning calorimetry (DSC) and thermogravimetric analysis (TGA) were performed using a NETZSCH STA 449F3 Jupiter thermal analyzer (Selb, Bavaria, Germany). The T_g_ was determined as the peak or midpoint of the heat capacity increase at the second heating cycle, and Tm as the endothermal peak in the DSC curve of the second heating cycle. From the TGA curves were obtained the temperature required for a 5% weight loss (T_5%_), which is representative of the onset of decomposition, the temperature of maximum decomposition rate (T_deg_), and the amount of residual mass at 600 °C.

Copolymer films were prepared by the solvent casting method, from polymer composition samples dissolved in methylene chloride. Polymer samples were prepared at 10% (*w*/*v*) concentration. HA was added to the polymer solution at 20% (*w*/*v*) concentration, with respect to the contents of the polymer composition. Polymer compositions were blended by a magnetic stirrer at room temperature and cast on the glass flat surface by a manual film applicator, then allowed to evaporate in a desiccator, to obtain a homogeneous film.

The tensile test was performed using an Instron-3366 universal mechanical testing machine. Samples were cut into rectangular pieces, about 0.15 mm thick, 10 mm wide, and 30 mm long. The sample gauge length was 10 mm and the testing speed was 1 mm/min. The results were taken as an average of five tests.

The SEM were obtained on a Hitachi TM300 electron microscope (Hitachi, Tokyo, Japan).

Water contact angle (WCA) measurements, between the polymer films and MilliQ water drops, were measured by the sessile drop method, on a “Tracker” automatic drop shape analyzer (Teclis-Scientific, Civrieux-d’Azergues, France), for drops of 100 s age, at a temperature of 25 °C and about 100% relative humidity. All measurements were repeated at least four times for each polymer sample.

The water absorption studies were performed in MilliQ water (pH = 7.00), at 25 °C. The amount of fluid absorbed through time was monitored gravimetrically. Dry 20 mm × 20 mm film samples (approximately 40 mg) were immersed in 50 mL of water and at regular time intervals were removed, dried with filter paper, weighed, and placed in the same bath. The water absorption (q) was calculated by the following equation:

q = (m_t_ − m_0_)/m_0_ (1)

where m_t_ is the weight of the absorbed polymer film at the time of measuring, and m_0_ is the initial weight of the polymer film [50].

## 3. Results

### 3.1. The Structure of Linear and Branched Pegylated Copolylactides

In this work, a number of MPEG-containing amphiphilic copolylactides were obtained; PLLA blocks were either linear (for copolylactide 1) or branched containing BHP monomer units (for copolylactides 2–4). The polymerization of L-lactide was carried out at 140 °C, as recommended in [51]. By systematic variation of composition and molecular weight of the polylactide block, a series of oligomeric amphiphilic copolymers, with varied ratios of PEG and PLLA molecular units, were prepared. The synthesized branched pegylated copolylactides are waxy substances, soluble in CH_2_Cl_2_, DMSO, and THF.

**Figure 2:**
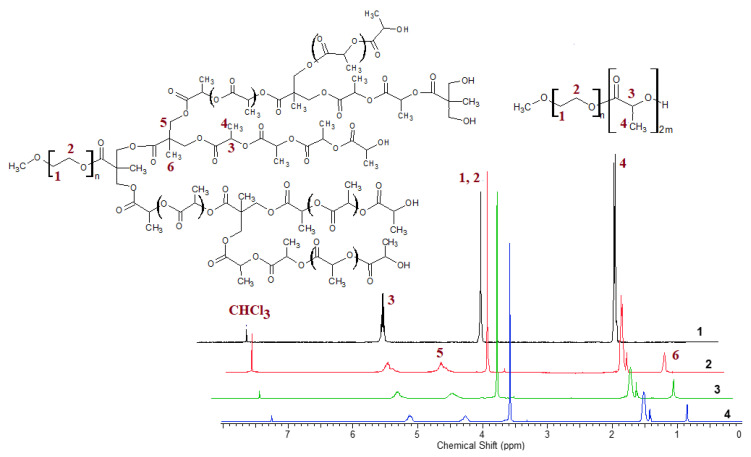
^1^H NMR spectra of synthesized polymers. The indices at the spectra correspond to the designations of the polymers in Table 1.

The structure of the synthesized linear and branched pegylated copolylactides was confirmed by ^1^H NMR spectroscopy; the assignment is shown in Figure 2 and is consistent with the literature data [28,44,52].

The incorporation of the MPEG was confirmed by the ^1^H NMR spectroscopy for both the linear and branched copolylactides. The ratio of incorporated PEG and PLLA units was determined from the respective ^1^H NMR signal intensity: thus, PEG units show characteristic signals at 3.56–3.70 ppm, that can be used to quantify their fraction, by comparison with the signals of the (>CH–) group of the lactide monomer units located at 5.1 ppm [47].

The synthesized copolylactides, the molecular parameters of which are summarized in Table 1, were characterized by narrow unimodal MWDs, with dispersity indices in the range of 1.17–1.22. According to the GPC data, the values of the average molecular weights of the copolylactides were in the range 8400–12,400 (Table 1), and this is probably somewhat on the low side, since polystyrene standards were used to calibrate the columns. It is important that in polymers changed either structure of PLLA block (linear or branched), or ratios of PEG/PLLA units (30:70 or 50:50), or molecular weight of the PEG block (1900 or 5000Da). The agreement between the calculated and experimental ratios of PEG and PLLA blocks was satisfactory. A decrease in the yield of pegylated copolylactides, with an increase in the amount of BHP in the reaction mixture, was observed (Table 1).

### 3.2. The Properties of PLLA Films Plasticized with Branched Pegylated Copolylactides Filled with Hydroxyapatite

Prior to the examination of PLLA plasticized with copolymers, the DSC analysis of pure polymers was performed. The obtained data are listed in Table 2.

All the studied copolylactides had a wide bimodal peak at 38.7–54.7 °C, the maxima of which corresponded to the Tg of the PEG and PLLA blocks. Along with this, pronounced monomodal peaks, corresponding to the Tm of PEG and PLA, were observed. While the Tm peaks of PEG and PLLA did not change significantly for all samples, the temperature of Tg peaks for PEG and PLLA blocks increased with their molecular weight.

Five PLLA films, plasticized with 10 wt% of different copolylactides, were obtained by the solvent casting technique. Furthermore, five composite films, containing 20% by weight of hydroxyapatite, were obtained using polymer matrices with 10 wt% of different copolylactides.

**Figure 3:**
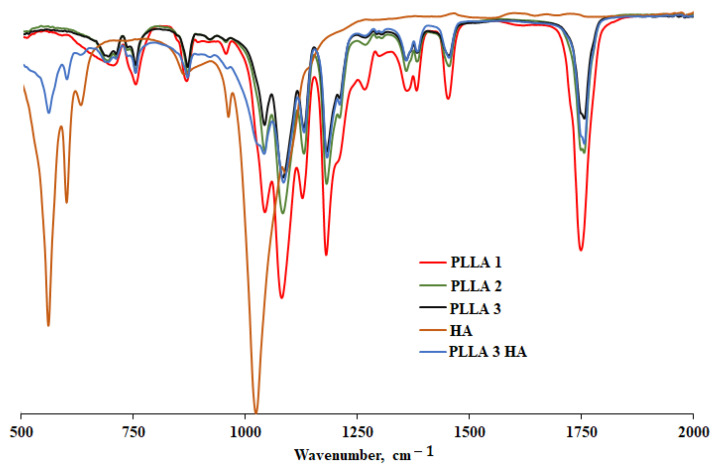
IR spectra of unfilled and hydroxyapatite-filled copolylactide films.

The IR spectra of the PLLA 1, PLLA 2, and PLLA 3 samples, shown in Figure 3, contained all the signals characteristic of copolylactides [53], and were close to the IR spectra of the PLLA 4 and PLLA 5 samples. At the same time, filling with hydroxyapatite lead to the appearance of characteristic absorption bands in the 500–700 cm^−1^ region.

**Figure 4:**
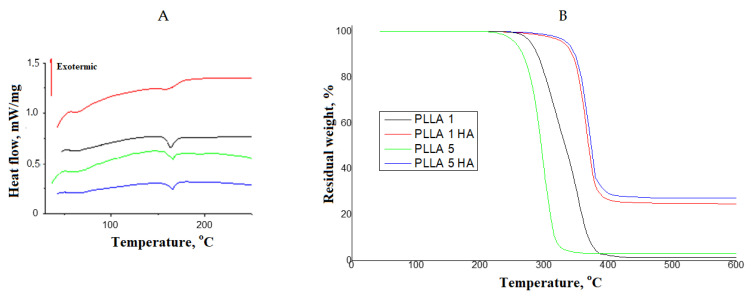
Representative thermal analysis data for composite samples: DSC curves (**A**) and TGA curves (**B**).

The thermal transitions of the prepared PLLA blends were assessed by DSC experiments. Typical DSC thermograms of PLLA 1, PLLA 1 HA, PLLA 5, and PLLA 5 HA composites (second heating run), are given in Figure 4. The designations of the obtained samples of polyester composites, as well as their compositions and thermal properties, are given in Table 3.

The glass transition temperature (T_g_) and the melting point (T_m_) of the PEG and PLLA phases, were evaluated for the polyester composites shown in Table 3. The samples of composite films had a wide glass transition temperature range, that sits between the glass transition temperatures of pure PLLA and pegylated copolylactides. The monomodal nature of DSC in the temperature range corresponding to the relaxation transition (glass transition), indicates good compatibility of pegylated copolylactides and pure PLLA for all studied compositions [54].

The addition of copolylactides to PLA only slightly changed the T_g_ and T_m_ values of neat PLLA; a correlation was observed between the molecular weights of the copolylactide blocks of PLLA and the Tg of the PLLA films plasticized by them. Addition of HA slightly increased the glass transition values of the PLA composites, perhaps due to a reduction of the chain segment mobility of the polymeric amorphous phase around the HA particles [55].

To study the thermal stability of the obtained films, thermogravimetric analysis (TGA) was performed. The experimental data are summarized in Table 3.

One can observe that HA containing samples underwent thermal decomposition in a single step, ranging from approximately 260 to 400 °C, which correlates well with values reported elsewhere [56,57,58]. Interestingly, the films plasticized by linear copolylactide 1 showed the highest T_5%_ value (Table 3), but faster decomposition, which was reflected in a lower T_deg_ value. In the same case, the addition of copolylactides 2–4 neither caused significant changes in T_5%_ (for films without HA), nor did they remarkably reduce the thermal stability (for films containing HA). The temperature of the maximum decomposition rate for films plasticized with branched copolylactides was generally close to the temperature of the maximum decomposition rate of neat PLLA films, only the use of copolylactide 4 with the largest polylactide block led to a slight excess of T_deg_ over this value of neat polylactide.

The addition of 20% HA to the PLLA matrix improved the thermal stability of the polymer, as can be seen in Figure 4. A rise in the T_5%_ and in the T_deg_ was observed for all composite samples (Table 3), suggesting an insulating effect of HA incorporation. We did not observe an increase in the degradation rate of the polymer matrix with the presence of the HA, as was stated in [55].

One can also observe that the residual masses for samples without HA, were relatively low, and progressively increased with the molecular weight of copolylactides up to 3.1%. For compositions with 20% HA, since the filler is thermally stable at high temperatures and can also act as a heat barrier, it could enhance the formation of char after thermal decomposition. Thus, the residual weights of samples containing hydroxyapatite were higher than those of samples without hydroxyapatite.

**Figure 5:**
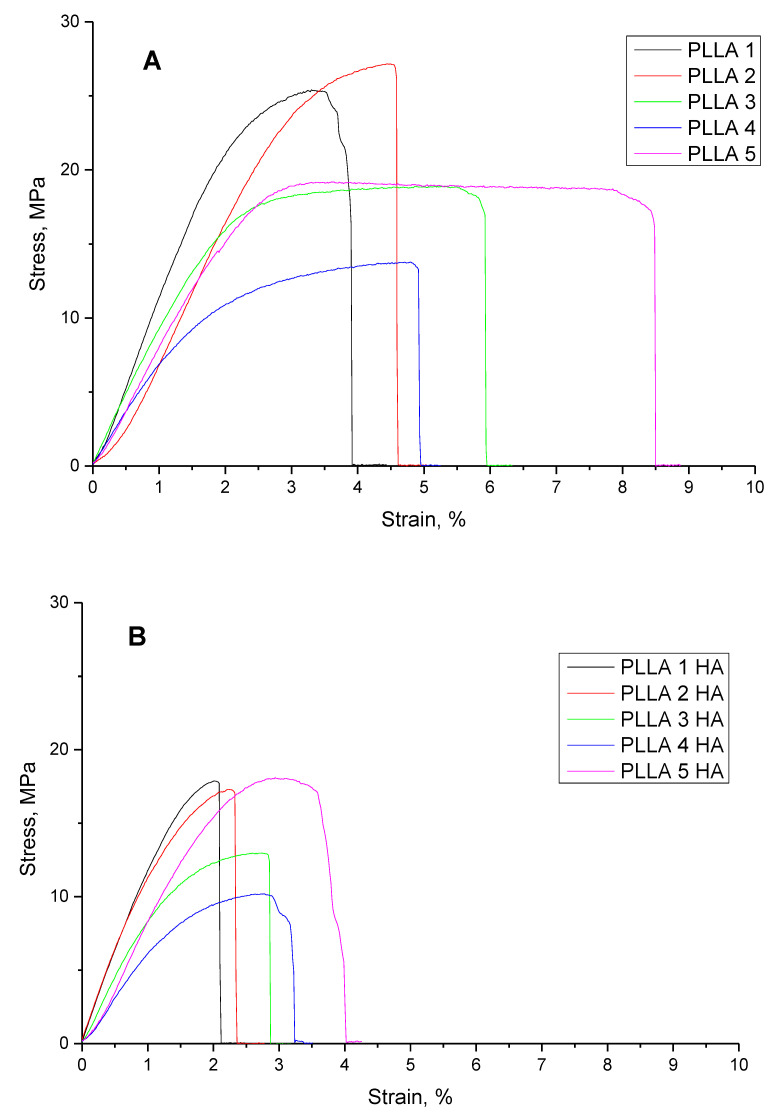
Tensile test curves for PLLA films without (**A**) and with (**B**) addition of 20 wt% of hydroxyapatite.

The tensile curves of PLLA films plasticized with copolylactides 1–4, as well as films of composite materials containing HA, are shown in Figure 5, and specific data are also given in Table 4. Pure PLLA 1 film showed mechanical properties of a hard but brittle material, with values of tensile modulus (E_t_) of 1290.8 MPa, tensile strength at break (σ_b_) of 25.4 MPa, and elongation at break (ε_b_) of 4.3% (Figure 5, Table 4).

The tensile modulus for blends of PLLA with copolylactides was lower than for pure PLLA. At the same time, plasticization of PLLA with linear copolylactide 1 led to mixtures with the tensile modulus only slightly lower than that of unplasticized PLLA. The presence of copolylactides with a branched PLLA block in mixtures, as a rule, led to a decrease in the tensile modulus of the polymer mixture. This effect decreased with increasing molecular weight of the copolylactides.

The filling of polyester films with hydroxyapatite had almost no effect on the elastic modulus of composite materials; however, the tensile strength and elongation at break decreased markedly. The low sensitivity of the elastic modulus of polyester materials to filling with hydroxyapatite indicated high compatibility of the matrix and filler. At the same time, it can be assumed that the interface between the polyester matrix and the filler (hydroxyapatite) becomes the center of the formation of defects and cracks under high mechanical loads. The decrease in the tensile strength of polyester materials when filled with hydroxyapatite, apparently, limits the achievable ultimate deformation corresponding to the rupture of composites.

The addition of copolylactides tended to plasticize the PLA film, resulting in a significant increase in flexibility and a decrease in tensile strength, as noted in various previous studies. Nevertheless, we found that the addition of linear copolylactide 1 caused only a slight increase in the value of elongation at break, which contradicts previously published data [59]. The yield point is observed only for copolylactides with high molecular weight branched PLLA blocks. Since the stage of plastic deformation is due to the parallel alignment of PLLA polymer chains, the presence of bulky high-molecular weight branched PLLA blocks increases the mobility of the polymer chains.

Typically, the addition of hydroxyapatite to PLLA in a 20:80 mix ratio, resulted in a decrease in ductility in all samples. However, the elongation at break for films plasticized with copolyesters was always higher than for unplasticized PLLA. Here, copolylactide 4 is of particular interest: the values of elongation at break for films plasticized with it were two times higher than for unplasticized films, regardless of the presence of HA in the film.

Tensile strength values for films plasticized with copolylactides were slightly lower than for pure PLLA films; at the same time, the PLLA 5HA sample plasticized with copolylactide 4 showed a tensile strength value characteristic of the unplasticized sample. This may be due to the good miscibility of the PLLA macromolecules and the plasticizer, as well as to the fact that HA particles are able to interact with the polar groups in branched PLLA polymer blocks [60,61]. As different polar groups, such as -OH, -CO, -CH_2_-O-CH_2_-, and -COOH, appear in the copolylactide polymer chains, they can react or interact with the -OH groups in commercial HA, as demonstrated by Zhang et al. [62].

**Figure 6:**
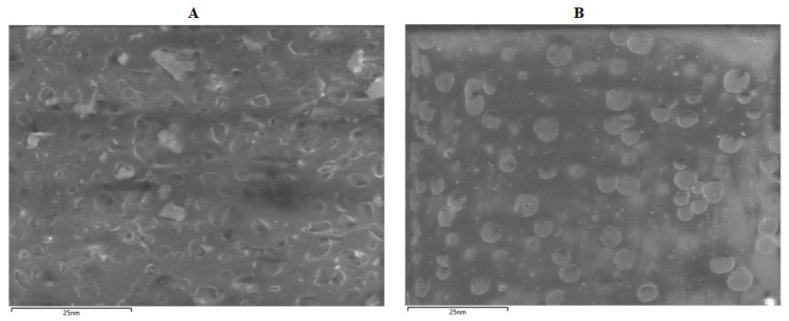
SEM of a polymer matrix (sample PLLA5)—(**A**) and a composite obtained by filling of the matrix with hydroxyapatite (sample PLLA 5 HA)—(**B**).

The SEM data indicate the compatibility of the copolylactide matrices with hydroxyapatite. The SEMs of the PLLA5 and PLLA 5 HA samples, shown in Figure 6, were typical for all obtained composite materials, and indicate a uniform distribution of the filler in the matrix volume. On the other hand, the pronounced surface roughness of the mixture of branched copolylactide 4 and PLLA (sample PLLA5) may be due to microphase separation of hydrophilic PEG fragments and hydrophobic lactide residues.

The hydrophilicity of the surface of the material is considered as a paramount requirement for its use in living organisms. Various studies have shown that wettability plays a key role in determining the ability of a polymeric material to serve as a scaffold for bone osteointegration [63,64]. However, the hydrophobicity of pure PLLA puts a limit on its compatibility with tissues and body fluids, and requires reduction. In this work, the PLLA hydrophilization was achieved by introducing hydrophilic polyethylene oxide fragments and a hydrophilic filler, i.e., hydroxyapatite. As can be seen in Table 4, the introduction of polyethylene oxide blocks and filling with hydroxyapatite led to a decrease in the water contact angle. Apparently, the value of water contact angle for the obtained composite materials is an integral characteristic of intermolecular interactions between hydrophilic and hydrophobic blocks in the composition of macromolecules, as well as hydrophilic polyethylene oxide blocks with hydroxyl groups on the surface of hydroxyapatite, used for filling. It can be assumed that if the main contribution comes from the interactions of the hydrophilic polyethylene oxide blocks with the hydroxyl groups on the surface of hydroxyapatite, then the surface layer is enriched with the hydrophobic polylactide fragments, which leads to an increase in the water contact angle, as was observed in the case of filling with PLLA3. Nevertheless, in the vast majority of cases, the contribution of hydrophobic interactions of polylactide fragments and displacement of hydrophilic polyethylene oxide fragments to the surface, is predominant, which leads to the desired effect of reducing the water contact angle.

**Figure 7:**
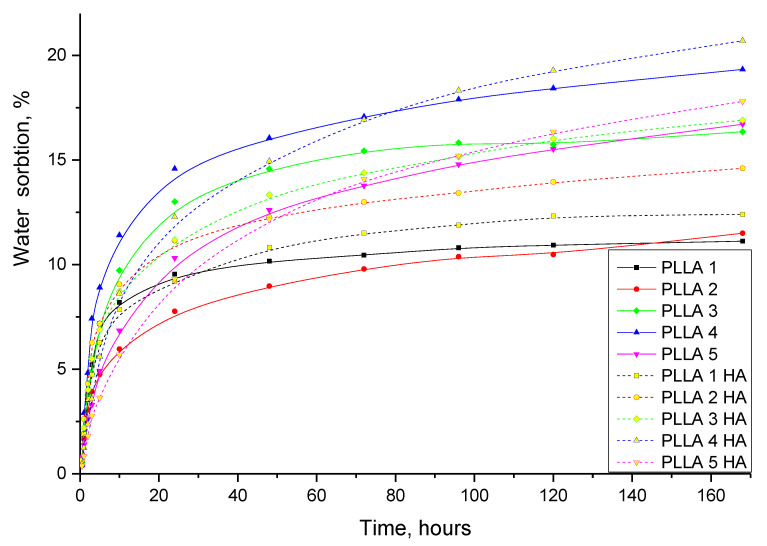
Kinetic curves for water absorption by different PLLA film samples.

The WCA observation can be correlated with the data on water absorption by polymer films shown in Figure 7. It is well known that the wetting and water absorption can play an important role in the release of a drug from the polymer matrix into an aqueous solution [65]. It is known that upon degradation of PLLA, the hydrophilicity of the polymer increases and the weight of the polymer decreases. Since these processes can occur simultaneously with water absorption, a relatively short time of 1 week was used to evaluate water absorption, in which polymer degradation processes do not have a large effect on the weight of the samples [66,67].

The amount of water absorbed by plasticized PLLA films is generally higher than by non-plasticized ones. The water absorption of all films steadily increased with time as ever deeper layers of the material became available for water. The water absorption of the films increased along with the rise in the mole fraction of hydrophilic pegylated fragments in the compositions of the branched copolylactides, as expected. The PLLA films not plasticized with copolylactides, as expected, absorbed water at the lowest rate. Among plasticized films, the sample containing linear copolylactide 1 had the lowest water absorption rate. Films plasticized with branched copolylactides showed the highest water absorption rates. Samples containing copolylactide 3, with the lowest molecular weight, showed the fastest water absorption. This may be due to different accessibility of the interior of the polymer matrix for water. Whereas linear copolylactides form fairly dense polymeric structures, branched copolylactides resulted in looser, permeable polymeric structures. In addition, low molecular weight copolylactide 3 may have the lowest adhesion to the polymer matrix and be removed from the polymer with water. All samples containing hydroxyapatite absorbed water faster than samples without hydroxyapatite of the same polymer composition, perhaps due to the hydrophilic nature of hydroxyapatite. In this case, the same tendences are observed as for films not containing HA: films plasticized with linear copolylactides absorb less water than films plasticized with pegylated branched copolylactides. Low molecular weight copolylactides absorb more water than films containing high molecular weight copolylactides.

It is known that, unlike linear polylactides, the hydrolytic degradation of which proceeds in bulk, the hydrolytic degradation of branched polylactides has a surface nature [68]. Although the determination of the dynamics and kinetics of the biodegradation of the obtained composite materials will be the subject of subsequent studies, it can be assumed that the rate of weight loss due to hydrolysis will hardly differ from that characteristic of linear PLLA, since the minimum content of the latter was 72 wt% at the maximum content of branched pegylated copolylactides (10 wt%) (Table 3).

## 4. Conclusions

It was established that the polymerization of L-lactide, catalyzed by tin (II) 2-ethylhexanoate in the presence of both BHP and BHP ester with MPEG, leads to the formation of branched pegylated copolylactides with a narrow molecular weight distribution. It was shown that the introduction of PEG fragments into linear and branched copolylactides leads to a decrease in the glass transition temperatures and melting points, as compared to PLLA. The resulting pegylated copolylactides were compatible with PLLA when blended, and formed films with higher tensile strength, lower strength, and lower tensile modulus. The introduction of 10 wt% of the synthesized pegylated copolylactides into PLLA did not have a significant effect on the melting points and the glass transition temperatures of the resulting interpolymer mixtures, but lead to a decrease in the water contact angle and increased water absorption. Additional hydrophilization of PLLA interpolymer blends and pegylated copolylactides can be achieved by filling with hydroxyapatite. In this case, the strength and ultimate elongation in tension decreased. Since HA-containing scaffolds are very important for medicine, the presented study will provide more information about their behavior and may lead to their wider use.

## Figures and Tables

**Table 1:** Molecular parameters for PEG-co-PLLA copolylactides (BHP was used in an equimolar amount with respect to PEG units).

Copoly-Lactide	PEG Mn	Yield	PEG/PLLA Units		M_n_ *	M_n_ **	M_w_/M_n_ **
			Calculated	Measured *			
1	1900	92	30:70	31.2:68.8	8640	8400	1.17
2	1900	87	30:70	30.3:69.7	9540	8700	1.22
3	1900	84	50:50	51.5:48.5	5030	4600	1.14
4	5000	85	50:50	51.8:48.2	13,220	12,400	1.20

*—Obtained from ^1^H NMR spectra of the polymers; **—Obtained by GPC.

**Table 2:** Characteristics of polymer materials used.

Polymer	PEG		PLLA	
	T_g_	T_m_	T_g_	T_m_
PLLA	-	-	60.5	163.8
Copolylactide 1	45.3	48.7	46.8.	153.6
Copolylactide 2	45.1	47.6	46.2	154.0
Copolylactide 3	45.4	39.7	38.4	148.5
Copolylactide 4	52.1	57.9	54.7	158.0

**Table 3:** Thermal properties of PLLA films.

Sample	Copolylactide	Composition (%)			T_g_ (°C)	T_m_ (°C)	T_5%_ (°C)	T_deg_ (°C)	Residual Mass (%)
		PLLA	Copo Lylac Tide	HA					
PLLA 1	-	100	0	0	61.2	164.2	279.3	361.4	2.1
PLLA 2	Copolylactide 1	90	10	0	59.8	166.4	280.7	295.3	1.9
PLLA 3	Copolylactide 2	90	10	0	56.2	165.1	254.1	299.1	2.2
PLLA 4	Copolylactide 3	90	10	0	60.3	166.7	257.2	307.5	2.7
PLLA 5	Copolylactide 4	90	10	0	60.7	165.3	255.8	314.7	3.1
PLLA 1 HA	-	80	0	20	64.3	158.8	330.5	368.1	24.8
PLLA 2 HA	Copolylactide 1	72	8	20	64.4	163.0	350.8	360.2	25.2
PLLA 3 HA	Copolylactide 2	72	8	20	63.4	162.3	332.4	365.6	27.7
PLLA 4 HA	Copolylactide 3	72	8	20	59.1	163.7	332.7	364.3	26.2
PLLA 5 HA	Copolylactide 4	72	8	20	63.1	165.8	336.6	369.7	27.4

**Table 4:** Properties of composite polyester materials: tensile modulus (E_t_), elongation at break (ε_b_), tensile strength (MPa) (σ_b_), and water contact angle (WCA) on different PLLA substrates.

Sample	E_t_ (MPa)	ε_b_ (%)	σ_b_ (MPa)	WCA (°)
PLLA 1	1290.8	4.3	25.4	93.3
PLLA 2	1076.1	5.1	27.4	76.8
PLLA 3	951.1	6.0	18.9	71.9
PLLA 4	672.3	5.4	13.9	88.5
PLLA 5	897.0	8.9	19.1	86.6
PLLA 1 HA	1295.0	2.4	17.9	75.9
PLLA 2 HA	1116.2	2.7	17.3	71.5
PLLA 3 HA	798.5	3.3	13.0	75.8
PLLA 4 HA	691.2	4.0	10.2	66.1
PLLA 5 HA	1042.4	5.9	18.1	66.4

## Data Availability

The data presented in this study are available on request from the corresponding author (the obtained initial data are fully presented in the article).
